# Comparative Analysis of Analog and Digital Chest Tube Drainage Systems Using a High‐Fidelity 3D‐Printed Neonatal Chest Model

**DOI:** 10.1002/ppul.71090

**Published:** 2025-04-11

**Authors:** F.‐X. Anzinger, T. J. Hashagen, P. Palaniappan, A. Lindner, M. Riboldi, J. Gödeke, O. J. Muensterer

**Affiliations:** ^1^ Department of Pediatric Surgery Ludwig‐Maximilians‐University of Munich | LMU Munich Germany; ^2^ Ludwig‐Maximilians‐University of Munich | LMU, Chair for Experimental Physics Munich Germany

**Keywords:** 3D printing, analog drainage system, chest model, chest tube drainage system, digital drainage system

## Abstract

**Background:**

In recent years, digital chest tube drainage systems have been introduced. Limited studies address their benefits and risks in pediatric patients, particularly neonates. This study compares a three‐chamber chest drainage system with a digital system using a high‐fidelity 3D‐printed model.

**Methods:**

We conducted direct measurements and 3D‐printed model tests with both systems at different suction pressures (−1 to −20 cmH_2_O) to assess the actual pressures. The effects of siphon and automatic flushes in the digital system were also studied.

**Results:**

At −20 and −10 cmH_2_O, significant differences were found between the digital and analog systems in direct and model measurements. The analog system became unreliable below −10 cmH_2_O. For the digital system, most measurements remained within the set pressures, with outliers up to −30 cmH_2_O due to regular flushing.

**Conclusion:**

This experimental study evaluates the suitability of digital drainage systems for the pediatric and neonatal populations. Our model demonstrated reliable simulation of thoracic conditions, making it a useful tool for pre‐clinical testing where patient testing may be limited. Both systems yielded satisfactory results at −20 and −10 cmH_2_O, but the digital system showed greater flexibility, maintaining pressures as low as −5 cmH_2_O. The analog system was consistent but less adaptable, which may limit its use in dynamic situations. The digital system's ability to simulate more flexible scenarios offers potential clinical advantages, though further investigation is needed to assess its impact on neonatal safety. The increase in suction during flushing may pose a risk for neonatal patients.

**Abbreviations**
3D: three‐dimensional
cmH_2_O: centimeters of water pressure
CT: computed tomography
IQR: interquartile range
MV: mean value
Pm: measured pressure
Ps: set pressure
SD: standard deviation
Traditional three‐chamber chest drainage system: analog drainage system

## 1 Background

The chest is a closed compartment that adheres to the principles of physics [1]. For nearly a century, three‐chamber water‐seal systems with external suction have been used to treat pneumothorax and postoperatively drain the thoracic cavity [2, 3]. However, these systems present certain drawbacks. Air leak measurement is subjective, and pressure settings are influenced by the system's position relative to the patient [2, 3]. Digital chest tube drainage systems have been developed to quantify air leaks and fluid volumes while allowing suction settings between −1 cmH_2_O and −100 cmH_2_O [4]. The pressure is monitored and adjusted by feedback mechanisms and regular tube flushes prevent obstructions.

Several randomized controlled trials and observational studies have compared the efficacy of digital drainage systems in adults [5, 6, 7, 8, 9], and children [10]. However, the results have been inconsistent, and studies addressing the benefits and risks in pediatric or neonatal populations are limited. Additionally, there is a scarcity of data regarding the suction accuracy and potential sources of error in both digital and analog drainage systems.

This study aims to compare analog and digital chest tube drainage systems using a self‐designed high‐fidelity 3D‐printed chest model that corresponds to a neonatal thorax. Our objective was to investigate the functionality of different drainage systems in vitro eliminating the confounding factors present in biological systems, to provide objective data on potential risks of using external suction in pediatric patients.

## 2 Study Design and Methods

The study consisted of two parts. First, direct measurements were conducted on a digital drainage system and compared to those of an analog system. The drainage's tube was directly connected to a pressure sensor. Second, we repeated the measurements on a 3D‐printed pediatric chest model as a surrogate. Drainage system and tube connected to a pressure sensor were inserted in the model's pleural cavity to simulate and simultaneously measure suction. We utilized a digital chest tube drainage system (*Medela Thopaz*+ [4]). For comparison, we used a traditional three‐chamber chest tube drainage system with external suction (*Argyle Thoraseal III* [11]). Furthermore, we investigated the behavior of the digital system when the pressure was set to −1 cmH_2_O as an alternative to clamping the drainage before removal, a practice still observed in some institutions.

Ethical Considerations: Institutional Review Board (IRB) approval was not required for this study, as it was conducted entirely in vitro using a chest model and did not involve human or animal subjects.

## 3 High‐Fidelity 3D‐Printed Chest Model

The 3D‐printed model used in this study was specifically developed for this investigation to meet the particular requirements of simulating pediatric thoracic conditions. The model was validated in terms of its suitability and functionality within the present study. Unlike other studies in this field, we focused on testing the accuracy and identifying potential sources of error when applying external pressure to a model, sized to replicate a neonatal chest [12, 13, 14, 15].

**Figure 1:**
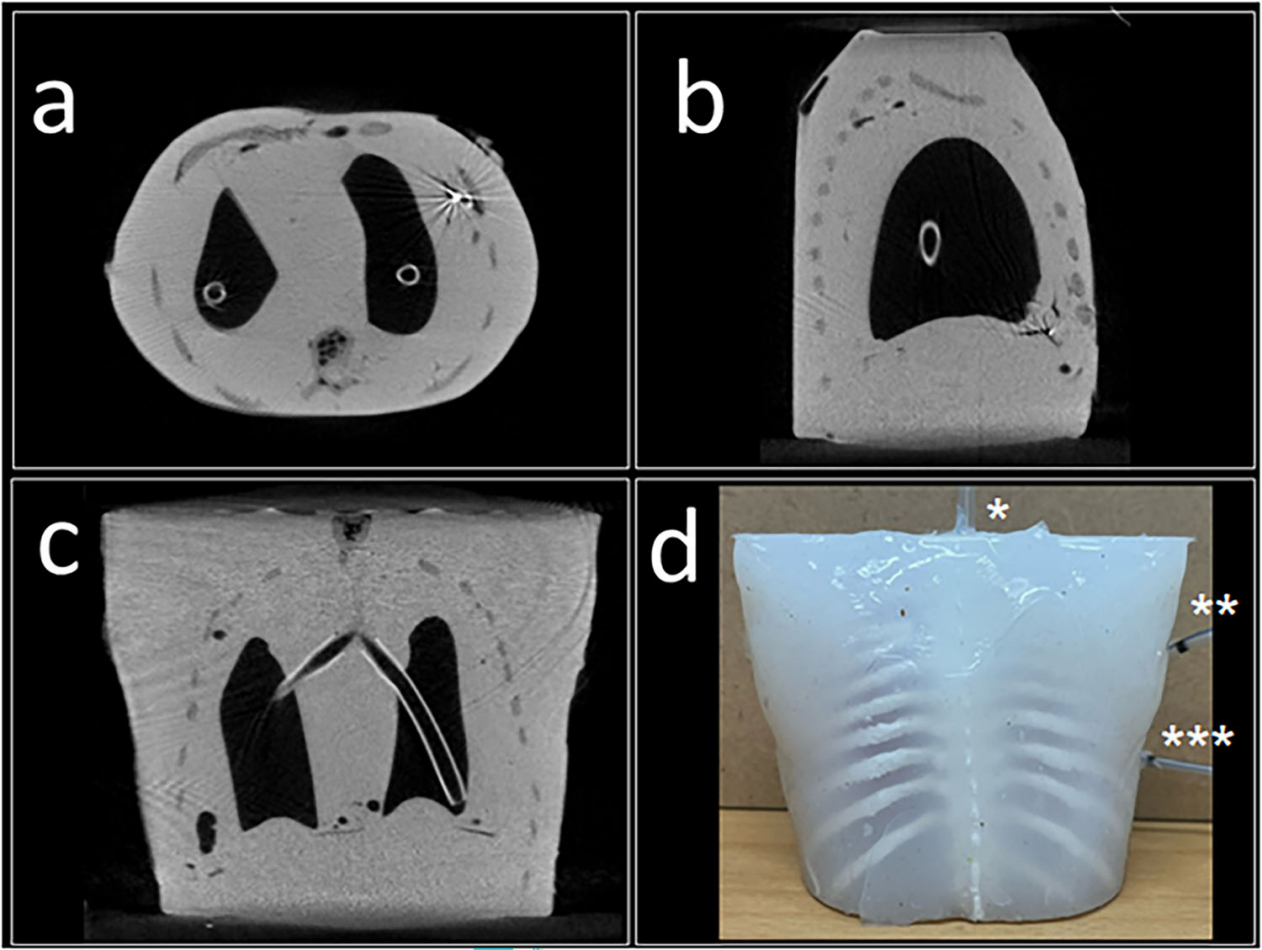
The high‐fidelity 3D‐printed model; (a) CT scan Axial plane of the model, (b) Sagittal plane, (c) Coronal plane, (d) Picture of the model with the tubes attached. [Color figure can be viewed at wileyonlinelibrary.com]

A silicone and plastic model, including an outer layer, two high‐fidelity lung phantoms, a trachea and a ribcage was designed and 3D printed. Ribcage and molds created from adult CT scans were downsized to 25%. By initially filling the lung phantom with gel that was later removed, we generated two separate inflatable hollow lungs. The ribcage was printed using polylactide to ensure both resistance and flexibility corresponding to physiologic conditions. The ribcage was enveloped in layers of silicone of different toughness mimicking pleural cavity and chest wall. Tubes were inserted into the artificial pleural cavity: one connected to the pressure sensor (***) and the other to the drainage system (**). An additional tube was placed in the artificial trachea (*) to insufflate both lungs. Breathing was simulated using a syringe, which was pushed and pulled regularly. One lung was punctured to allow airflow into the pleural cavity, simulating a pneumothorax. A picture of the model is shown in Figure 1d.

## 4 Two Drainage Systems

The *Medela Thopaz* + (Medela Medizintechnik GmbH & Co, Eching, Germany [4]) is a digital chest drainage and monitoring system. It provides sub‐atmospheric pressure application to the patient's chest, which can be regulated to a set pressure from −1 to −100 cmH_2_O. Pressure is measured and adjusted in a feedback loop. The display provides real‐time data and graphs of air leak and fluid collection. The tube is regularly flushed, every 5 min or when a siphon is detected [4]. A siphon in a chest tube refers to the unintended flow of fluid or air through the tube due to a pressure difference, which can occur when the height of the water or fluid column in the drainage system is greater than the pressure in the pleural cavity. This can cause a reversal of fluid or air flow, which might lead to complications. A siphon can create negative pressure that might cause further lung collapse or tissue damage. Therefore proper positioning and maintaining the correct height of the water seal chamber are obligatory. The *Argyle Thoraseal III* (Cardinal Health Germany 507 GmbH, Norderstedt, Deutschland [11]) is a traditional three‐chamber chest drainage system, including a collection chamber, water seal, and suction chamber to regulate external suction by applying a certain water level [11].

## 5 Measurements

A highly sensitive piezoresistive silicon pressure sensor (*Honeywell, ABP2MRRT060MDAA5XX*, Honeywell Sensing & Safety Technologies, 830 East Arapaho Road, Richardson, TX 75081 [16]) with built‐in calibration for temperature effects and accuracy errors was utilized. Accuracy errors included non‐linearity, repeatability, and hysteresis, with a total error of ±1.5% full scale span = 0.9 mbar. The readout electronics were designed and assembled using an Arduino Uno (A microcontroller board developed by Arduino s.r.l., Monza, Italy) electronic device. The real‐time monitoring system was developed using graphical user interfaces in Arduino IDE (Integrated Development Environment) and LabVIEW (Laboratory Virtual Instrument Engineering Workbench, National Instruments, Austin, Texas, USA) [16].

Initially, direct measurements were conducted using the digital drainage system at settings of −1, −5, −10 and −20 cmH_2_O (see Table 2). The sensor was connected directly to the drainage system for real‐time measurements. Subsequently, the model was used with the same settings. Comparable measurements were attempted with the analog drainage system, calibrated for archiving exactly −10 and −20 cmH_2_O [11]. Different water levels were used to imitate settings of −5 to −20 cmH_2_O (see Table 1).

**Table 1:** Medians and IQRs from all measurements (analog drainage system), Wilcoxon signed‐rank test was used for comparing the data to the set values (below each median) and comparing the direct to the phantom data (last column).

Analog system set pressure (Ps)	Direct measurement measured pressure (Pm1) *p* values *(Ps/Pm1)*	3D model measured pressure (Pm2) *p* values *(Ps/Pm2)*	Comparative *p values (Pm1/Pm2)*
−5 cmH_2_O	−9.11 (−10.9 to −8.96) < 0.001	−9.11 (−9.11 to −8.96) < 0.001	0.0296
−6 cmH_2_O	−11.05 (−11.05 to −10.90) < 0.001	−10.16 (−10.16 to −10.01) < 0.001	< 0.001
−7 cmH_2_O	−11.80 (−11.95 to −11.35) < 0.001	−10.90 (−11.05 to −10.75) < 0.001	< 0.001
−8 cmH_2_O	−11.35 (−11.50 to −11.35) < 0.001	−11.35 (−11.35 to −11.20) < 0.001	0.0026
−9 cmH_2_O	−12.55 (−12.55 to −12.40) < 0.001	−12.25 (−12.25 to −12.10) < 0.001	< 0.001
−10 cmH_2_O	−12.55 (−12.85 to −12.25) < 0.001	−10.90 (−10.90 to −10.75) < 0.001	< 0.001
−20 cmH_2_O	−22.26 (−22.7 to −21.96) < 0.001	−21.44 (−22.11 to −20.76) < 0.001	< 0.001

The impact of a siphon in the tube was measured in both drainage systems through series of 5‐min measurements. A siphon was achieved by bending the tube in U position and filling it with 40 mL of water. Additionally, the regular flush mechanism of the digital system was studied at a 10 ms temporal resolution.

For each pressure setting, 10 sets of multiple measurements were conducted, each lasting 30 min at a sampling rate of one per second, resulting in 1.800 data points per set. The data was than down sampled for statistical evaluation. Medians and IQRs were calculated for different measurements. The flush mechanism of the digital system was monitored in real time using a higher sampling rate (10 ms). Data acquisition and visualization were performed using LabView runtime standalone application after conversion of units through the Arduino IDE code.

## 6 Results

### 6.1 Analog Drainage System

**Figure 2:**
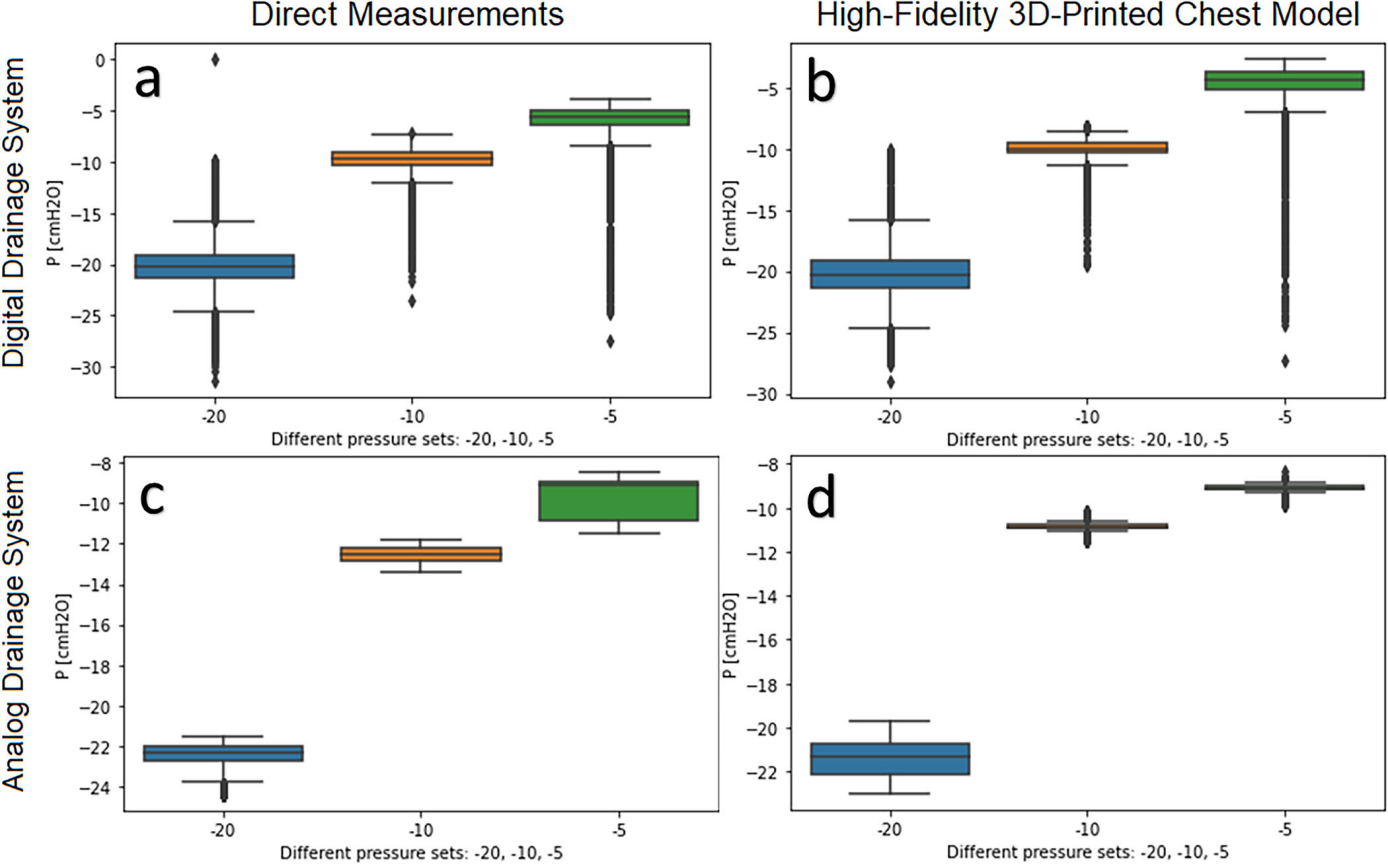
Medians and IQRs from single measurement in direct measurements (a, c) and the high‐fidelity 3D‐printed chest model (b, d) in digital drainage system (a, b) and analog drainage system (c, d). [Color figure can be viewed at wileyonlinelibrary.com]

For the settings of −0, −10 cmH_2_O and below, the measured pressure (Pm) differed from the set pressure (Ps) in both direct measurement and chest model. The median difference between the pressure settings was 3.67 cmH_2_O when compared to the direct measurement (Pm1) and 3.02 cmH_2_O when compared to the 3D phantom model (Pm2). Pm values were higher for −20 and −10 cmH_2_O settings in both direct measurements and the model by 0.82 cmH_2_O and 1.65 cmH_2_O, respectively, as shown in the box plots for the analog system (Figure 2c,d). Medians and IQRs are presented in Table 1. The model measurements were consistent with the direct measurements for settings between −10 and −20 cmH_2_O. However, settings below ‐10 cmH_2_O were inaccurate in our experiments, yielding Pm values ranging from −9.11 to −12.55 cmH_2_O for both direct and model measurements (Table 1). When filling the water chamber with only half the volume required for −10 cmH_2_O, we observed pressures of −10.9 to −8.96 cmH_2_O (see Table 1, Figure 2c,d).

### 6.2 Digital Drainage System

A maximum system error of −1 cmH_2_O was assumed for each pressure setting. The digital system maintained pressure settings effectively for both direct and model measurements, with the exception of −1 cmH_2_O. The results were consistent for settings of −10 and −20 cmH_2_O in direct and model measurements with a difference of 0.30 cmH_2_O for −10 cmH_2_O and 0.14 cmH_2_O for −20 cmH_2_O. However, for settings of −5 cmH_2_O and below, measured pressure in the model was 1.35 cmH_2_O lower than in direct measurements.

**Figure 3:**
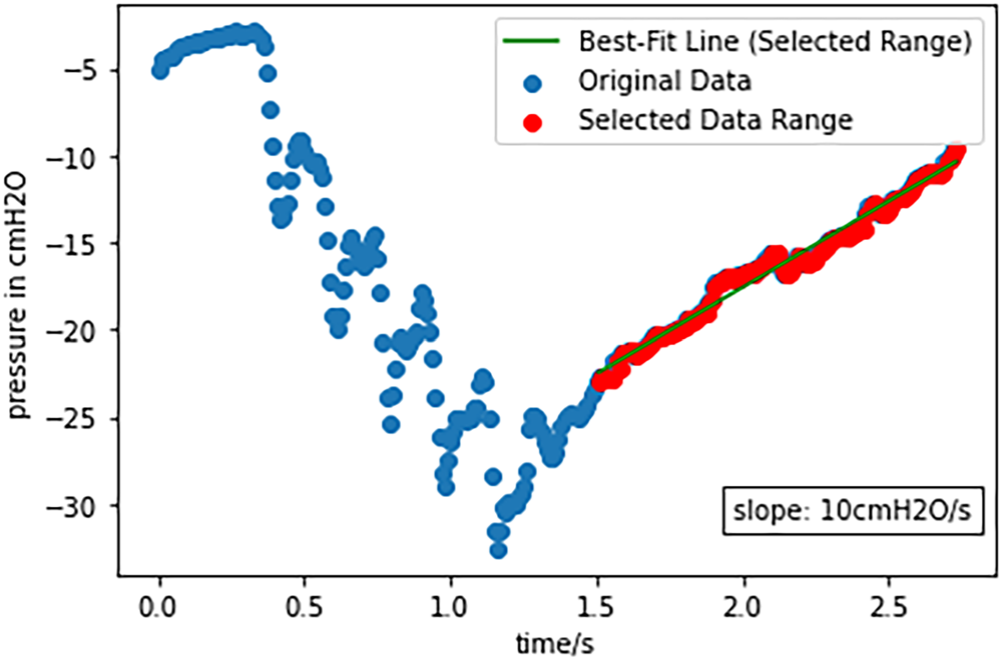
Flush at −5 cmH_2_O pressure set at 10 ms sample rate. [Color figure can be viewed at wileyonlinelibrary.com]

The vast majority of data points fell within the expected range for −20, −10, and −5 cmH_2_O settings, as illustrated in the box plots (Figure 2a,b). However, outliers up to −30 cmH_2_O were attributed to the tube flushing, which occurred every 5 min. The flush sequence is shown in Figure 3 for a −5 cmH_2_O setting, sampled at a 10 ms rate. High suction pressure was rapidly achieved within 1.2 s following a brief pressure drop, and it took 2 additional seconds for the pressure to return from −25 to −5 cmH_2_O at a rate of 10 cmH_2_O/s.

### 6.3 Siphon Effect

When a siphon was created, the digital system initiated constant flushing until the fluid was evacuated from the tube and pressure returned to −10 cmH_2_O. Stabilization to −15 cmH_2_O took approximately 80 ± 2 s, and reaching −10 cmH_2_O took 120 ± 2 s. The siphon's initial height of 55 cm corresponded to an additional pressure of 55 cmH_2_O. The siphon height was manually adjusted to 40, 30, 15, and −50 cm (siphon in opposite direction).

### 6.4 Reduction to −1 cmH_2_O

At −1 cmH_2_O, pressure increased due to flushes but returned to baseline after 180 s. Median pressures were −2.09 (−4.93 to −0.90) cmH_2_O in direct measurements and −0.15 (−2.39 to 0.30) cmH_2_O in the model, indicating a 108% increase and a 85% decrease (see Table 2).

**Table 2:** Medians and IQRs from all measurements (digital drainage system), Wilcoxon signed‐rank test was used for comparing the data to the set values (below each median) and comparing the direct to the phantom data (last column).

Digital system (Ps)	Direct measurements measured pressure (Pm1) *p* values *(Ps/Pm1)*	3D model measured pressure (Pm2) *p* values *(Ps/Pm2)*	Comparative *p* values (Pm1/Pm2)
−1 cmH_2_O	−2.09 (−4.93 to −0.90) < 0.001	−0.15 (−2.39 to 0.30) < 0.001	< 0.001
−5 cmH_2_O	−5.68 (−6.42 to −4.93) < 0.001	−4.33 (−5.08 to −3.73) < 0.001	< 0.001
−10 cmH_2_O	−9.71 (−10.31 to −9.11) < 0.001	−10.01 (−10.31 to −9.56) 0.73	0.009
−20 cmH_2_O	−20.17 (−21.36 to −19.12) < 0.001	−20.31 (−21.36 to −19.12) < 0.001	0.18

### 6.5 Statistical Evaluation

Statistical analyses were performed to assess differences between the analog and digital system measurements across various pressure levels for both direct and model measurements. Due to the measured outliers, our data was not normally distributed. Wilcoxon signed‐rank test was used for comparing the data to the set values of each pressure setting.

Direct measurements: Significant differences were observed between analog and digital system for the pressure settings: −5, −10, −20 cmH₂O (*p* < *0.001*).

Model measurements: Significant differences between the two systems were also noted for −5, −10, −20 cmH₂O (*p* < 0.01). Both direct and model measurements differ significantly between the analog and digital systems across all tested pressures, with strong significance for lower pressure settings.

Comparison of direct and model measurements: Significant differences were observed at settings of −1, −5, −10 cmH₂O (*p* < 0.01) in the digital system. A combined analysis averaging direct and model measurements within each system showed statistically significant differences across all pressure levels: −5, −10, −20 cmH₂O (*p* < 0.001) (See Table 3).

**Table 3:** Combined medians and IQRs from digital system and analog system with *p* values comparing digital and analog drainage system via Wilcoxon ranked sign test merging direct and phantom measurements.

Set pressure (Ps)	Digital system direct measurements (Pm)	Digital system 3D model (Pm)	Analog system direct measurement (Pm)	Analog system 3D Model (Pm)	Comparative *p* values
−1 cmH_2_O	−2.09 (−4.93 to −0.90)	−0.15 (−2.39 to 0.30)	—	—	—
−5 cmH_2_O	−5.68 (−6.42 to −4.93)	−4.33 (−5.08 to −3.73)	−9.11 (−10.9 to −8.96)	−9.11 (−9.11 to −8.96)	< 0.001
−10 cmH_2_O	−9.71 (−10.31 to −9.11)	−10.01 (−10.31 to −9.56)	−12.55 (−12.85 to −12.25)	−10.90 (−0.90 to −10.75)	< 0.001
−20 cmH_2_O	−20.17 (−21.36 to −19.12)	−20.31 (−21.36 to −19.12)	−22.26 (−22.7 to −21.96)	−21.44 (−22.11 to −20.76)	< 0.001

## 7 Discussion and Interpretation

This is the first experimental, systematic study to assess the function of digital versus analog drainage systems for thoracic surgery in neonates. To date, few studies have explored the use of digital drainage systems in the treatment of pediatric patients, with limited attention to potential risks stemming from anatomical differences between adults and children. Also, there are so far no experimental studies that objectify the suitability of digital systems for the pediatric and neonatal population. Our study aimed to fill this gap by investigating the functionality of the different drainage systems in a high‐fidelity 3D‐printed model, eliminating confounding biological factors [12, 13].

Da Silva Costa Jr. et al. were among the pioneers in describing postoperative air leaks in a *Medela Thopaz+* device [17] in pediatric patients. Their findings suggested that the use of a digital drainage system simplifies the decision‐making process during the postoperative period, reducing the risk of misinterpreting air leak data. However, they emphasized the need for further research, given the small sample size of 11 patients under 14 years.

Alam et al. divided a cohort of 100 pediatric patients with thoracic empyema into two groups of 50. One group was treated with an analog drainage system, while the other received the *Medela Thopaz DTDS* [18]. 40% were 10 years or younger. Patients treated with the digital system experienced significantly shorter durations of air leak (5.34 vs. 7.16 days), tube placement (7.44 vs. 10.44 days), and postoperative hospital stay (10.16 vs. 14.76 days), along with fewer postoperative complications. In their randomized study, the authors demonstrate the superiority of the treatment. Recognizing the right time for drain removal can therefore lead to shorter treatment. However, as the study only covers thoracic empyema, the results cannot be directly applied to aseptic thoracic surgery.

Pérez‐Egido et al. conducted an analysis of the postoperative safety and effectiveness of digital drainage systems in pediatric patients compared to analog drainage systems [19]. Their study comprised 26 patients, with 13 receiving treatment with a digital system and 13 with an analog system. Notably, the median age of the patients was 18 months, significantly lower than in other studies. In the digital drainage group, mean treatment duration was 1.69 ± 0.6 days, compared to 5.38 ± 4 days in the analog drainage group. Additionally, the average hospital stay was shorter (5.69 ± 2.7 vs. 7 ± 4.7 days), and the number of postoperative radiographs was significantly reduced (2.8 ± 1.1 vs. 6.23 ± 5.2). The study demonstrates how digital drainage systems can provide an objective measurement of air leakage that can lead to early chest tube removal and less postoperative radiographs. All three studies emphasize the benefits of facilitating decision‐making based on objectifiable and reliable data through a digital drainage.

In our experimental study, we conducted a comparison between an analog and a digital drainage system using a high‐fidelity 3D‐printed chest model to investigate the different drainage systems in vitro. The statistical analysis indicates substantial and consistent differences between the analog and digital systems at all pressure settings. We observed significance across both direct and model measurements.

At suction pressures of −20 and −10 cmH_2_O in the analog drainage system, the measured values consistently exceeded the set pressure, with an median difference of 1.65 cmH_2_O in our model. Attempting to achieve accurate suction pressures below −10 cmH_2_O with the analog system proved unsuccessful. Suction pressures below −10 cmH_2_O were unreliable in the analog drainage system utilized in this study. In the context of treating newborn patients, reducing the water level below −10 cmH_2_O did not translate into corresponding lower pressure levels. This inaccuracy could translate into certain risks for patients, particularly in neonates. In terms of accuracy our measurements suggest that digital drainage systems outperform the analog ones in the lower pressure range. While the digital system theoretically has the capacity to achieve pressures as low as −1 cmH_2_O, the peak pressures generated during flushes prolonged the time required to return to the low set pressure, resulting in elevated pressures. Specifically, when set to −1 cmH_2_O, median suction pressures ranged from −2.09 cmH_2_O in direct measurements to −0.15 cmH_2_O in the model. Setting the digital system to −1 cmH_2_O may be a valid alternative to clamping the drainage before removal as there remains a minimum negative suction. This practice would not endanger the patient as the act of clamping a tube could theoretically do by allowing the formation of a tension pneumothorax in the worst case.

At suction pressure settings of −20 and −10 cmH_2_O, both direct and model measurements yielded similar results for the digital drainage system. However, at −5 cmH_2_O and −1 cmH_2_O the direct measurements exhibited higher amplitudes with significant difference compared to the model. This discrepancy can be attributed to an increased likelihood of pressure loss through small leaks that become apparent with lower constant suction. An alternative interpretation is that the flushing of the tube can be detected with more accuracy in the direct measurement than in the model due to the sturdiness of the system.

When studying the behavior of the digital system, it is important to consider the programmed flushing cycles, which increase the suction in 5‐min intervals. Flushing the tube was the primary cause of deviations from the set pressure when using the digital system in our study. Although the flushing itself took approximately 3 s, the pressure increases were up to −30 cmH_2_O. The implications of these findings for the delicate lung tissue of infants and newborns remain unclear. It is conceivable that high peak pressures can pose a theoretical risk to patients. Practitioners should be aware that their patients are exposed to higher pressures at regular intervals.

Upon contacting the manufacturer, we learned that the regular increases in suction were due to the automatic flushing as well as deliberate flushes of the system when the device detects an obstruction. A prospective observational study showed that 36% of drains occlude after cardiac surgery. Up to 86% of these occlusions occur within the patient, invisible to the medical staff [20]. Flushing the tube therefore has a certain value in the therapy. Further developments are currently on the way to reduce the risk of obstruction, for example a hydrogel polymer coating to minimize the risk of clot formation. Future studies need to quantify the clot formation risk to decrease the frequency and the amplitude of the tube flushing in infants. In contrast, the analog drainage system did not exhibit the pronounced fluctuations induced by tube flushing. In our study there were no significant fluctuations in the suction pressure of the analog system.

When a siphon is introduced, the digital drainage system continuously flushes until the siphon is neutralized within a matter of minutes, a capability lacking in the analog system. The absence of mechanisms to counteract siphons in analog systems means that fluctuations, such as those caused by regular adjustments and flushing in digital systems, cannot occur. Consequently, pressure applied to the patient's chest may experience significant increases or decreases, depending on the siphon's height. Particularly concerning are instances of positive pressure, which can pose life‐threatening risks [1, 21]. Additionally, high suction levels, particularly in pediatric patients, can be perilous [1, 8]. Mitigating siphon formation can be achieved by utilizing rigid plastic tubing to minimize bending and ensuring regular monitoring of tube positioning [1, 11]. Preventing siphon formation is paramount in traditional water seal drainage system management [1, 2, 21].

### 7.1 Clinical Implications and Safety Considerations

Clinicians should be aware that digital systems, while superior in accuracy and functionality at lower suction pressures, may expose patients to short periods of elevated pressure during automatic flushing. This characteristic should be considered when treating neonates, whose lung tissues are particularly sensitive to pressure changes. There is no data from which we know that flushing is associated with an increased risk of infection. Nevertheless, thoracic seeding of infectious material is theoretically possible. Further studies are needed to answer this question. Adjusting the frequency and amplitude of flush cycles or enhancing system design to reduce clotting may be areas for future innovation.

To use digital chest drains safely, doctors and nurses must continue to be trained in the use of these devices. Knowledge of the dangers and benefits is the most important step in ensuring patient safety.

Our results validate the use of high‐fidelity 3D‐printed chest models as a reliable method for testing chest tube drainage systems. The strong correlation between direct and model measurements support the use of this model for further studies assessing system behavior and safety.

The limitations of this study include its in vitro nature, which means that the findings are theoretical and may not fully translate to clinical practice. Additionally, only two commercially available drainage systems, one analog and one digital, were tested. Other systems may behave slightly different than the ones used in this study.

#### 7.1.1 Conclusion

The traditional analog drainage system was less flexible in handling and more error‐prone in the lower suction range. The digital drainage system, on the other hand, was more accurate in lower suction settings, while also producing stable suction levels when dealing with a syphon. However, this increase in suction pressure during flushing may theoretically pose a certain, unquantifiable risk for neonates. Additional studies need to address this issue in more detail. The close alignment of suction pressure values obtained from both direct and model measurements affirm the validity of our model [12, 13, 14, 15].

Further investigations are warranted to better understand the safety considerations and potential hazards associated with the treatment of pediatric patients in general, as well as neonates in particular. Clinicians must weigh these factors when selecting a drainage system for pediatric and neonatal patients.

### 7.2 Take‐Home Points

#### 7.2.1 Study Question

How do analog and digital chest tube drainage systems compare in terms of accuracy, safety, and functionality when tested using a high‐fidelity 3D‐printed neonatal chest model?

#### 7.2.2 Results

The digital drainage system demonstrated greater accuracy in maintaining set pressures, especially at lower suction levels, while analog systems showed inconsistencies below −10 cmH_2_O. The automatic flushing in digital systems caused temporary pressure spikes that may pose risks in neonatal care.

#### 7.2.3 Interpretation

While digital chest tube drainage systems offer superior precision and adaptability, the pressure fluctuations during automatic flushing require attention, particularly for neonatal patients. Awareness of these fluctuations and improvements in system design are essential for safer clinical application.

## Author Contributions

**F.‐X. Anzinger:** conceptualization, writing – original draft, methodology. **T. J. Hashagen:** conceptualization, investigation, methodology, software, formal analysis, data curation. **P. Palaniappan:** conceptualization, validation, visualization, writing – review and editing. **A. Lindner:** conceptualization, validation, resources, data curation. **M. Riboldi:** validation, supervision. **J. Gödeke:** conceptualization, writing – review and editing. **O. J. Muensterer:** conceptualization, supervision, project administration, writing – review and editing.

## Consent

All authors agree to be accountable for all aspects of the work in ensuring that questions related to the accuracy or integrity of any part of the work are appropriately investigated and resolved. The corresponding author, F.X.A. had full access to all the data in the study and had final responsibility for the decision to submit for publication.

## Conflicts of Interest

The authors declare no conflicts of interest.

## Data Availability

The data sets used and/or analyzed during the current study are available from the corresponding author on reasonable request.
